# Clot Accumulation in 3D Microfluidic Bifurcating Microvasculature Network

**DOI:** 10.3390/mi15080988

**Published:** 2024-07-31

**Authors:** Merav Belenkovich, Ruth Veksler, Yevgeniy Kreinin, Tirosh Mekler, Mariane Flores, Josué Sznitman, Michael Holinstat, Netanel Korin

**Affiliations:** 1 Faculty of Biomedical Engineering, Technion-Israel Institute of Technology, Haifa 32000, Israel; meravkr@campus.technion.ac.il (M.B.); kreinin@campus.technion.ac.il (Y.K.); tiroshmekler@campus.technion.ac.il (T.M.); sznitman@bm.technion.ac.il (J.S.); 2 Department of Pharmacology, University of Michigan Medical School, Ann Arbor, MI 48109, USA; marianen@med.umich.edu (M.F.); mholinst@med.umich.edu (M.H.); 3 Department of Internal Medicine, Division of Cardiovascular Medicine, University of Michigan Medical School, Ann Arbor, MI 48109, USA; 4 Department of Surgery, Division of Vascular Surgery, University of Michigan Medical School, Ann Arbor, MI 48109, USA

**Keywords:** thrombosis, microvasculature, hemodynamics, von Willebrand factor, Murray law, bifurcation, platelet

## Abstract

The microvasculature, which makes up the majority of the cardiovascular system, plays a crucial role in the process of thrombosis, with the pathological formation of blood clots inside blood vessels. Since blood microflow conditions significantly influence platelet activation and thrombosis, accurately mimicking the structure of bifurcating microvascular networks and emulating local physiological blood flow conditions are valuable for understanding blood clot formation. In this work, we present an in vitro model for blood clotting in microvessels, focusing on 3D bifurcations that align with Murray’s law, which guides vascular networks by maintaining a constant wall shear rate throughout. Using these models, we demonstrate that microvascular bifurcations act as sites facilitating thrombus formation compared to straight models. Additionally, by culturing endothelial cells on the luminal surfaces of the models, we show the potential of using our in vitro platforms to recapitulate the initial clotting in diseases involving endothelial dysfunction, such as Thrombotic Thrombocytopenic Purpura.

## 1. Introduction

Thrombosis, the pathological formation of blood clots inside blood vessels, lies at the core of a variety of cardiovascular diseases, which are one of the leading causes of worldwide mortality: from an increased blood pressure due to flow obstruction to embolic stroke caused by clots that migrate to the brain vasculature and halt oxygen supply to the brain. As presented by Rudolf Virchov back in 1856, in what is famously known as the Virchow’s triad, thrombosis is governed by three determinates: endothelial dysfunction (i.e., damage to the endothelial vessel wall layer), the hypercoagulability of blood, and hemodynamics—blood flow conditions. Thus, local changes in flow conditions, particularly near the vessel wall, can significantly control clot formation and growth [1,2]. In this context, the Wall Shear Rate (WSR), defined as the velocity gradient near the wall, is acknowledged as a well-known parameter that affects the mass transport and friction forces near the wall and, thus, regulates thrombosis processes and kinetics [3]. The WSR is geometry-dependent: it changes along different vessels in the human vascular system, with an average value of ~300 1/s, rarely rising above 1000 1/s in non-pathological conditions. Vessels containing irregular geometries may result in the development of complex flow patterns. For example, in partially obstructed diseased vessels, the WSR might increase by one or two orders of magnitude. These changes are considered to favor thrombosis [1,3]. Numerous studies have examined the effect of geometry changes on flow conditions [4,5,6,7], and specifically, the effect of the presence of bifurcations on the formation of thrombosis [5,7,8,9]. Martorell et al. showed the effect of flow recirculation on the expression of atherosclerotic and thrombotic biomarkers in coronary and carotid bifurcations [5]. Carrascal et al. presented a numerical study on the influence of blood clots in a bifurcating blood vessel where an increase in platelet activation in a bifurcated vessel occurred relative to a straight model [9]. While these studies focused on large arterial bifurcations [7], bifurcations in the microvasculature are also of great physiological relevance.

The microvasculature constitutes the vast majority of the cardiovascular system and is characterized by complex structures, typically including multiple bifurcations. Its structure also varies from organ to organ. The microvasculature plays a key role in numerous physiological and pathological processes, such as inflammation and thrombosis, as well as conditions that incorporate both. However, its microscale dimensions, with vessel sizes on the same scale as blood cells, pose a challenge for the computational modeling of blood transport and thrombosis processes [10]. Thus, such processes have been mainly studied using a variety of animal models. However, animal vasculature and blood flow therein are different from those of humans in terms of size, hemodynamics, protein expression, the immune system, and more. For example, conditions that are considered to be pathological in humans might be completely normal in mice [3,10].

As an in vitro alternative to animal testing, microfluidic devices, including organs-on-a-chip assays, have been a subject of great interest in general, as well as in the field of vascular thrombosis. Early perfusion-based thrombosis models consisted of straight collagen-coated channels, where the blood perfusion rate was adjusted to a physiological or pathological shear rate, depending on the blood vessel type and thrombus composition [11]. Later, photolithographic mold fabrication paved the way for more complex geometries, allowing for a more precise replication of the vascular structure and flow regime. For example, stenotic microchannels were utilized to model plaque occlusion in atherosclerosis [12,13]. The vascular biology aspect of these microfluidic devices was enhanced with the addition of endothelial cells, allowing for the study of platelet–endothelial interactions [14], as well as disease mechanisms [15].

Yet, thrombosis models of small blood vessels remain scarce, as the construction of artificial microvasculature becomes more challenging with the decrease in vessel dimensions. Tsai et al. presented a state-of-the-art microfluidic device as an in vitro hemolytic uremic syndrome model, consisting of an endothelial-cell-lined bifurcating design reaching 30 µm × 30 µm dimensions at the smallest channels. The endothelial cells were activated with a Shiga toxin and perfused with whole blood at different shear rates to examine the role of the von Willebrand Factor (vWF) in the pathology, as vWF is known to be a key molecule in thrombosis and platelet adhesion both on collagen and when endothelial cells are inflamed [16]. On a larger scale, Zheng et al. compared the vWF patterns of stimulated endothelial cells under the perfusion of whole blood from healthy volunteers and TTP patients, as well as vWF–platelet interactions in the presence and absence of ADAMTS13. Endothelial cells in this study were cultured in 100–1000 µm channels of various shapes and were induced to over-secrete vWF using the secretagogue phorbol myristate acetate (PMA) [17]. These two important studies only emphasize how little work is still available in this field, where microfluidic devices constitute a promising platform for recapitulating various pathologies in the human microvasculature and the development of novel therapeutic approaches. Nevertheless, even these state-of-the-art microfluidic models have a major drawback in spatially replicating microvessel networks—they are not all 3D in nature, as all microchannels are of the same height.

Cecil Murray (1926) was the first to recognize the relationship between the vascular organization and function, and address the work of blood against the friction of the vessel wall [18]. Murray deduced that, to achieve the maximal efficiency in the circulation (minimal work), the shear rate must be conserved between blood vessels. It is now well established that the shear rate changes along the blood circulation, but it remains an eligible approximation, which will be implemented and assessed in this work. However, microdevices replicating the vascular network that are of the same height cannot properly replicate the flow and the WSR associated with bifurcation in microvessel networks.

In this work, we present an in vitro model for clotting in bifurcating microvessels, with a focus on 3D bifurcations that are in line with the Murray law design. Using these models, we show the effect of bifurcations as sites of facilitated thrombus formation. Additionally, we also demonstrate the potential of using this platform to recapitulate the initial clotting in diseases that involve endothelial dysfunction, such as Thrombotic Thrombocytopenic Purpura (TTP).

## 2. Materials and Methods

### 2.1. Computational Fluid Dynamics (CFD)

CFD simulations were conducted in ANSYS fluent 2023 R1 using a laminar flow assumption. Fluid with a density of 1060 kg/m^3^ and a constant Newtonian viscosity of 3.5 cP was used to simulate blood. The boundary conditions included no-slip conditions on the channel wall and a uniform inlet velocity, calculated according to the channel dimensions and the given inlet flow rate. The targeted wall shear rate was 1000 1/s, thus, the inlet velocity was set to 3.3 mm/s, corresponding to a flow rate of 80 µL/min.

### 2.2. CFD-Based Design of the Microfluidic Device

**Figure 1:**
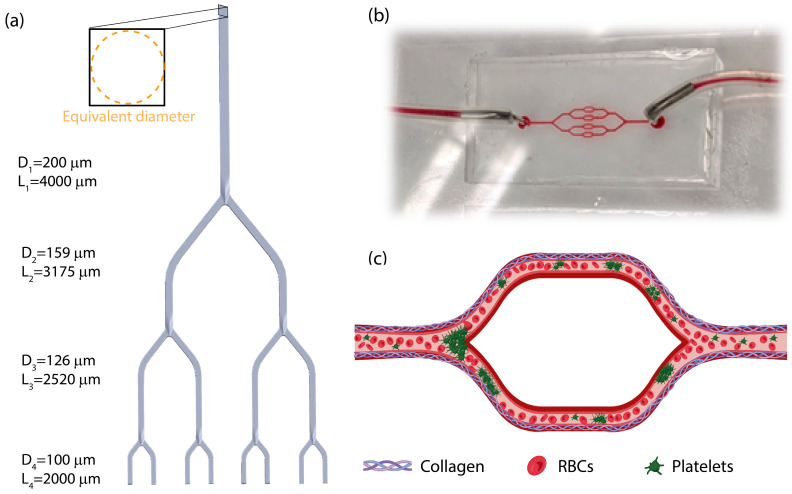
Murray’s Law for a bifurcating microchannel network design. (**a**) Illustration of microchannel cross-section and branching dimensions. (**b**) Image of the final model after fabrication perfused with blood. (**c**) Illustration of platelet-based clotting in a bifurcation microfluidic vascular injury model upon perfusion with blood (created with BioRender.com).

The 3D bifurcated structure was designed to mimic physiological branching inspired by the cerebral vascular system, while conserving the macro-scale network structure. Each channel equivalent diameter was used for the calculation of the cross-section dimensions of successive vessels according to Murray’s Law, as further described in the simulation section (Figure 1a). The model was designed in SOLIDWORKS. A microchannel mold was 3D printed using a Form3 printer with a clear resin V4 (Formlabs). The printed part was rinsed with isopropyl alcohol to remove non-cross-linked resin, then cured under UV light at 60 °C for 1 h.

The microfluidic devices were fabricated using standard soft lithography techniques from polydimethylsiloxane (PDMS) in a polymer cross-linker ratio of 10:1 (*w*/*w*) [19]. Inlet and outlet holes were created using a 1 mm biopsy punch. Glass slides were coated in a thin PDMS layer at a 5:1 ratio (*w*/*w*) using a spin-coater and baked at 60 °C overnight. To covalently bond the PDMS slab to the coated slides, both sides were exposed to an oxygen plasma, followed by baking at 60 °C for 1 h. For the final assembly of the microfluidic device, connectors with silicone tubing were inserted into the holes (Figure 1b).

### 2.3. Blood Perfusion Experiments

#### 2.3.1. Vascular Injury Model

The exposure of sub-endothelial matrix collagen during a disease-mediated injury of the blood vessel wall was imitated by coating the microfluidic device channels with collagen. The devices were first placed in a desiccator for at least 30 min to remove all air bubbles and assure the full coverage of the channel with the collagen solution. Then, 100 µL of 3 mg/mL Human Type I collagen solution (Vitrocol^®^, Advanced Biomatrix, Carlsbad, CA, USA) was mixed with 890 µL HBS (HEPES buffered saline, pH 7.2, 20 mM) and 10 µL NaOH (sodium hydroxide, 0.1 N) and manually perfused through the channel. Overnight incubation at 37 °C facilitated further cross-linking of the collagen fibers, followed by PBS washing of the channels to remove the excess collagen.

##### Human Blood

Whole blood was taken from healthy human volunteers and supplied by the Israeli National Blood Service (Rambam Medical Center Institutional Review Board (IRB) RMB-0413-21). A total of 9 mL blood was collected from each volunteer into a citrated blood collection tube.

##### Platelet Staining

To each blood tube, 0.5 μg/mL of DiOC_6_ (3,3′-Dihexyloxacarbocyanine Iodide, Thermo Fisher Scientific, Waltham, MA, USA) was added, a cell-permeant lipophilic dye for the staining of the mitochondria and other organelles of live cells, which is commonly used for platelet staining in thrombosis studies. The blood was then incubated at 37 °C for 30 min.

#### 2.3.2. Endothelial Cell Culture and Handling

##### Cell Culture and Microfluidic Cell Seeding

Primary Human Umbilical Vein Endothelial Cells (HUVECs, Lonza, Bazel, Switzerland) were cultured in growth medium (ECM, ScienCell, Carlsbad, CA, USA)-containing flasks to confluence (up to 7 days) and incubated in a 37 °C humidified environment with 5% CO_2_. Experiments were performed with cultures of passages 4–6.

Prior to seeding the cells in the microfluidic device, all device components were sterilized with UV light for 15 min in biological hood. The microdevice channels were coated with human fibronectin (100 μg/mL, Sigma-Aldrich, St. Louis, MO, USA) and stored at 4 °C overnight. Endothelial cells were seeded manually in the microdevice at a density of 4–6 × 10^6^ cells/mL and incubated for 2–4 h to allow the cells to settle at the bottom of the channel.

##### Endothelial Cell Stimulation

Following cell adhesion, the channels were slowly perfused with a fresh medium to clear the unattached cells before observation under a light microscope to ensure that the endothelial cells were relaxed and elongated. To recapitulate the TTP phenotype of propagated vWF elongation, vWF secretion from the endothelial cells was induced by infusing the microchannels with 50 ng/mL of phorbol 12-myristate 13-acetate (PMA) in serum-free medium and incubation at 37 °C and 5% CO_2_ for 40 min.

##### Immunofluorescence Staining

Cell post-seeding growth was observed under a confocal microscope, following 40 min of incubation at 37 °C with CellTracker™ (live staining, Invitrogen, Waltham, MA, USA). After cell stimulation, the ECs were fixated by incubation with 4% paraformaldehyde (PFA) for 15 min at room temperature and washed with PBS. 0.05% Triton™ X-100 (Sigma-Aldrich, St. Louis, MO, USA) was infused for 3 min to permeabilize the cell membrane prior to immune staining. Then, 1 h of incubation with 10% fetal bovine serum (FBS) was used as a blocking procedure to decrease the non-specific interactions of fluorochrome–antibody conjugates.

The endothelial cells’ shape was determined by staining the F-actin in the cell cytoskeleton with Alexa Fluor™ 568 Phalloidin (Invitrogen, Waltham, MA, USA) for 40 min.

vWF was stained with a rabbit origin Anti-vWF antibody (ab9378) at 1% dilution in 1% FBS following 1 h of incubation, and donkey anti-rabbit IgG NL493-conjucated antibody (cat. #NL006, R&D systems, Minneapolis, MN, USA) was used as a secondary antibody in 5% dilution in PBS, following 1 h of incubation.

Lastly, 4′,6-diamidino-2-phenylindole (DAPI) was used to stain the cell nuclei at a short incubation duration of 5 min. All staining procedures were performed at room temperature.

### 2.4. Experimental Setup

The microfluidic device was fixed onto the moving stage of an upright fluorescent microscope (Nikon SMZ25, Nikon Instruments Inc., Melville, NY, USA). Then, a 1 mL syringe of whole blood was connected to the inlet tubing and clasped onto a syringe pump (Braintree Scientific Inc., BS-8000, Braintree, MA, USA). The perfusion flow rate was set to 23 μL/min or 80 μL/min to achieve the desired shear rates of 300 1/s and 1000 1/s, respectively. Each experiment was conducted in a paired manner, with a control channel perfused at the same time and under the same conditions as the test channel. Blood perfusion was monitored in both channels simultaneously, under fluorescence, to follow the clot propagation within the channel for a duration of 11 min (Figure 1c). All blood perfusion experiments were performed within 4 h from blood collection to preserve the platelet activity.

### 2.5. Data Analysis

Images were automatically captured in time-lapse mode in both channels at 30 s intervals. Image analysis was performed by Matlab^®^ (R2019a). Due to patient-to-patient variability, the final image of the bifurcated experiment for each patient was used to define a patient-specific threshold. Using the resulting threshold, each image was binarized, and the pixels were counted to obtain the kinetics graphs for each patient. All experiments were repeated at least five times.

## 3. Results

### 3.1. Fluid Dynamics and CFD

According to Murray’s law, the hierarchical structure of the vascular system is essential for an effective supply of oxygen, allowing the conservation of the shear rate and minimizing the amount of energy required to overcome the friction between the blood and the vessel wall. When a vessel of diameter *d*_0_ bifurcates into two branches of diameters *d*_1_ and *d*_2_, the diameter relation should follow (Equation (1)):

(1) $${d_{0}}^{3}={d_{1}}^{3}+{d_{2}}^{3},$$

The microchannel was designed such that it maintained the diameter relation defined in Equation (1). While the microchannel was designed with a square cross-section, dimensions could be regarded with an equivalent diameter, where decreases in both their height and width with each bifurcation were in accordance with Murray’s law.

**Figure 2:**
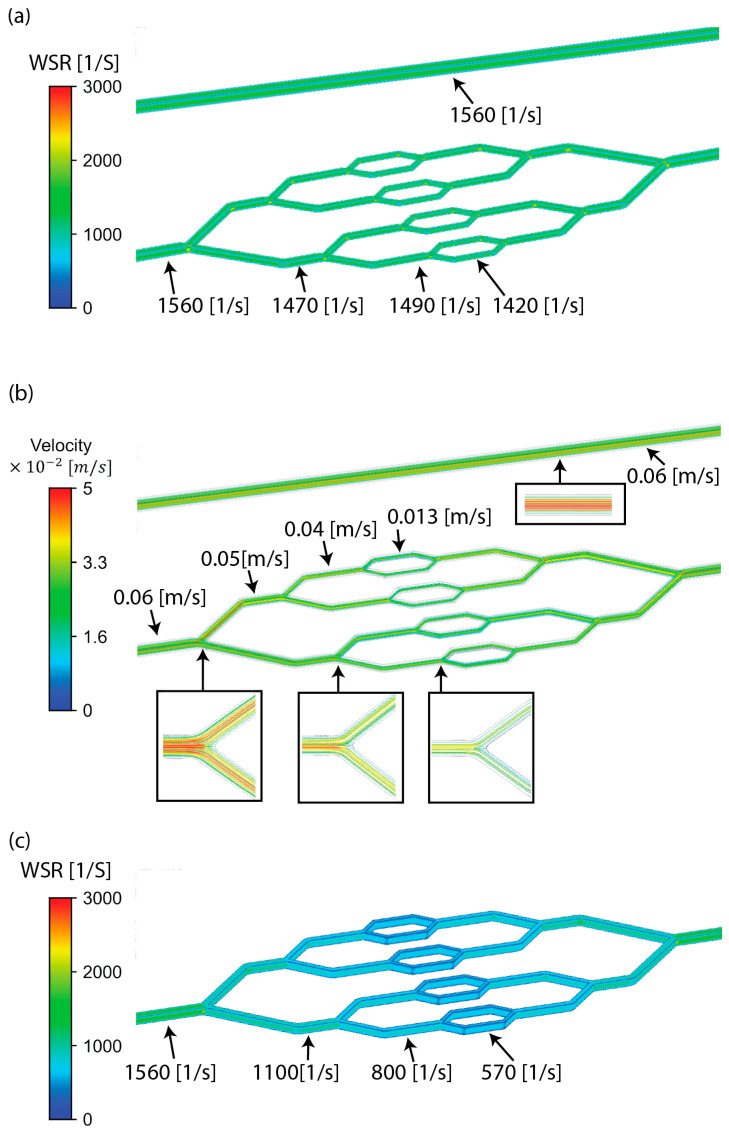
Computational fluid dynamics results. (**a**) WSR distribution for straight (**top**) and Murray’s-law-based model (**bottom**). (**b**) The flow field for straight (**top**) and Murray’s-law-based model (**bottom**). (**c**) WSR distribution in a similar model with a constant height. It can be seen that the WSR remains relatively constant throughout the Murray law bifurcated model in correspondence with the Murray’s law (Figure 2a—less than 10% change between bifurcating branches), while it changes dramatically in a similar model with an equal uniform height throughout the model.

The WSR distributions were examined via CFD simulations for the Murray’s-law-based model and for a flat model, with a uniform height throughout. It can be seen (Figure 2a) that a uniform WSR was achieved for the Murray-law-based bifurcated model, whose branching length also scaled similarly to Murray’s law, where *L*_1_ and *L*_2_ are the lengths of the corresponding vessel branches (Equation (2)):

(2) $$\frac{d_{1}}{d_{2}}=\frac{L_{1}}{L_{2}}=\sqrt[{3}]{2},$$

As noted, the CFD showed that the WSR in the Murray’s-law-based model remained constant, similar to the straight channel with similar dimensions. The velocity decreased when advancing across bifurcations, and as the flow was laminar (Reynolds ~1), the streamlines followed the bifurcation geometry (Figure 2b). However, in the flat model (Figure 2c), which had a constant height, the WSR decreased when advancing across bifurcations; thus, it cannot comply with Murray’s law.

### 3.2. Clotting in Bifurcating Microvessels

Small blood vessel occlusion (mainly arterioles and capillaries), following vessel wall lesions and platelet thrombosis, may be triggered by several factors. Here, we present two models for clotting in small vessels. The first represents the result of damage to the collagen layer of the vessel due to extended vascular injury, while the second recapitulates diseases that involve endothelial dysfunction such as TTP.

#### 3.2.1. Vascular Injury Model

The collagen coating of microchannels represents a worst case scenario of an extended vascular injury, where all endothelial cells are removed from a certain vascular region and the entire sub-endothelial matrix is exposed. This exaggeration is important for the initiation of the coagulation cascade, starting with platelet activation through collagen binding and ending in the formation of platelet-rich clots and the obstruction of microchannels.

To test the effect of bifurcation on thrombosis, the bifurcated model was fabricated and coated with collagen. As a control, a straight channel of the same cross-section and with the same WSR conditions as the inlet channel in the bifurcated model was also prepared and coated with collagen for blood perfusion. Fresh human blood was perfused at a flow rate of 80 µL/min for 11 min. Both microfluidic devices were of similar lengths and were perfused simultaneously.

**Figure 3:**
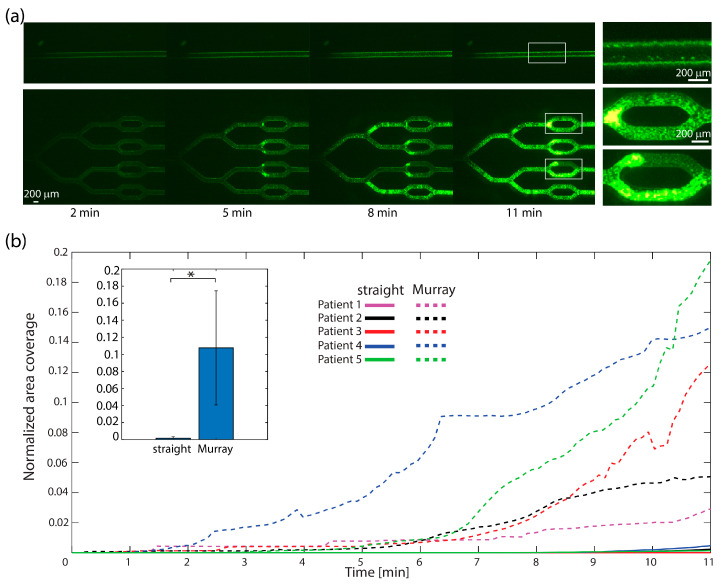
Clot propagation in collagen-coated bifurcated microchannel network. (**a**) Time-lapse fluorescent microscopy images of platelet accumulation in straight (**top**) and bifurcated (**bottom**) channel. While large platelet-rich clots are formed at the bifurcated model to the extent of obstructing the flow to certain branches, in the straight channel, the platelets merely bind the collagen to form small aggregates. (**b**) The graph represents the intensity changes over time by following the changes in fluorescence area coverage throughout the experiment. The signal in the straight channel remains stable and weak, while the signal in the bifurcated model increases with time. Inset bar graph presents the average of end results of 5 different patients. Significance was determined by unpaired Student’s *t*-test, * *p* < 0.05. Similar analysis of fluorescence intensity can be found in Figure S1, which shows similar behavior.

Figure 3a presents time-lapse images of a typical experiment and demonstrates the clot propagation in the bifurcated model (bottom), compared to the control straight channel (top). It can be seen that large platelet rich clots were formed within the bifurcated model to the extent of obstructing the flow in certain branches, while in the straight channel, the platelets merely bound to the collagen. In Figure 3b, the kinetics of clot propagation are represented by changes in the intensity level throughout the experiment; these changes are obtained by following the fluorescence area coverage of each image, normalized by the total area of the channel. It can be seen that the signal in the straight channel remained stable and minor, while the signal in the bifurcated model increased with time. These results indicate that more platelets adhered and aggregated in the bifurcated models, even though the shear rate was uniform throughout the model. These results imply that bifurcations are an important feature in the in vitro induction of thrombosis.

#### 3.2.2. Endothelial Dysfunction Model

Endothelial cells comprise the inner layer of blood vessels and have an important role both in the recreation of the human micro-environment and in mimicking vascular disease conditions. Here, we recapitulate in vitro pathologies that involve the dysfunction of the endothelial cells at the vessel wall. As proof of concept, we focus on TTP, a life-threatening thrombotic pathology of the brain capillaries, in which fast diagnostics is critical.

**Figure 4:**
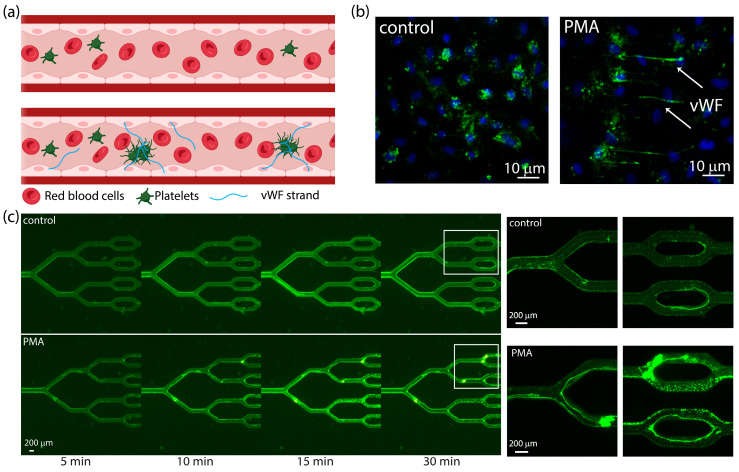
Thrombosis in PMA-stimulated HUVEC-seeded bifurcation microvessel network. (**a**) Illustration of clot formation in a microchannel seeded with cells stimulated to secrete vWF strands (created with BioRender.com). (**b**) Fluorescence images of HUVECs seeded in classic 2 mm × 80 µm straight channels, where cell nuclei were stained with DAPI (blue) and vWF was stained with FITC-conjugated antibody (green). Network-like structures are seen in the PMA-stimulated channel, indicating the secretion of vWF strands. (**c**) Time-lapse images of perfusion experiment of whole human blood in (**top**) control microchannel, where endothelial cells were not stimulated to secrete vWF, (**bottom**) test microchannel seeded with PMA stimulated endothelial cells. Platelets were stained in green.

In TTP, the lack of ADAMTS13 results in the uncontrolled elongation of vWF along the blood vessel walls, facilitating the formation of platelet-rich clots in small vessels (illustrated in Figure 4a). To mimic these disease conditions in animal models, ADAMTS13 knockout mice were insufficient—recombinant vWF was injected to induce thrombosis [20]. To replicate similar conditions in human in vitro models with blood from healthy individuals, endothelial cells were stimulated in microchannels using 50 ng/mL of PMA. Following stimulation, the cells were fixated and stained to evaluate the vWF expression pattern, as can be seen in Figure 4b. Without any stimulation, vWF should remain packed in secretory granules inside the cells—depicted in the control images as small dots in proximity to the cell nuclei. These dots were also detected in stimulated cells, but to a lesser extent, implying that the secretion was not complete. Nevertheless, in the stimulated cells, network-like structures surrounding the cells were found in abundance, describing a fast release of secretory granule contents. Similar to the previous section, we examined the effect of bifurcation on clot propagation.

HUVECs were seeded, PMA-stimulated, and fixated in the bifurcated model. The microfluidic devices were then perfused with whole blood from healthy volunteers at a shear rate of 300 1/s, while the platelets were fluorescently stained, and their adhesion was monitored under an upright microscope for 30 min. Figure 4c shows time-lapse images of a typical experiment comparing microchannels seeded with stimulated and unstimulated cells. While in the control vessels, we observed the mild random attachment of platelets, in the stimulated model, the platelets adhered to strands and aggregated to form clots. It is very possible that these strands were vWF secreted from the stimulated cells, binding the platelets and promoting their activation and aggregation. Although these are just preliminary results, they suggest that our cell-seeded microfluidic platform can replicate a key feature of thrombotic microangiopathies.

## 4. Discussion

In this work, we presented an in vitro microfluidic model for the recapitulation of thrombosis in bifurcating microvessels. We aimed to mimic human physiological conditions by designing a bifurcated structure with changing heights to follow Murray’s law of a conserved WSR between blood vessels. To examine the effect of bifurcation on thrombosis, we examined two models for clotting. The first recapitulated severe vascular injury that results in the entire exposure of the collagen layer, and the second recapitulated pathologies that involve endothelial dysfunction such as TTP. We designed 100–200 µm bifurcated microchannels inspired by a real microvascular structure, the cross-section of which decreased with each bifurcation according to Murray’s principle of minimum work. The CFD simulations supported that the wall shear rate was uniform throughout the model, verifying the applicability of Murray’s law to the microchannels’ dimensions. Although there are several works that have recapitulated the human microvasculature geometry and physiology to investigate vessel–blood cell interactions, and specifically in bifurcating microvessels, [4,10,16], to our knowledge, all previous microvessel models were made with the same height and, thus, do not conserve the Murray’s law. Here, for the first time, we applied changes in the cross-section with each bifurcation according to Murray’s principle of minimum work. This phenomenon has a direct effect on thrombosis, as flow is highly affected by local flow structures [21]. Few groups have previously looked at the effect of geometry and flow on clot formation. For example, Herbig and Diamond used a bifurcating microfluidic device to create a stagnation point flow and examined its effect on clotting, showing increased platelet-based clotting at the stagnation point [22]. However, their model did not mimic physiological microvessel bifurcations. Other groups that looked at physiological bifurcations, such as Tsai et al. [16] and Zheng et al. [17], did not focus on platelet-based clotting. Moreover, all of these models were designed with a uniform height and did not comply with Murray’s law.

Although human blood vessels have a circular geometry, the use of square or rectangular cross-section models is conventional in microfluidics to allow for planar-based microscopy imaging. Therefore, although this assumption causes some artifacts, mostly in the corners, the general flow behavior does not change, and the model can properly represent the flow dynamics in bifurcating microvascular networks.

Our perfusion experiment on collagen-coated microdevices demonstrated, through the fluorescent staining of platelets, that thrombosis was significantly increased at the bifurcated channels, compared to that in a straight microchannel of similar dimensions. These results are in compliance with in vivo observations. For example, using cranial intravital microscopy in well-established mouse models of congenital thrombotic thrombocytopenic purpura, Adili et al. observed that clotting was enhanced at sites of arteriole bifurcations [20].

Additionally, we showed that our model can be used to mimic various microvascular diseases and investigate vessel–blood cell interactions. As a proof-of-concept, we recapitulated the TTP pathology by seeding endothelial cells in our microdevices and stimulating them with PMA to secrete vWF. When we perfused human blood inside the cell-lined microchannels, we demonstrated that the platelets attached to the stimulated cells in strands and formed aggregates, while the adhesion to unstimulated cells was minor. This is compatible with the results presented by Tsai et al. [16], who used Shiga toxin for endothelial stimulation in a microvascular model. Although these results are still preliminary, they establish the platform potential to recapitulate TTP’s abnormal vWF manifestation and coagulation using blood from healthy volunteers, as well as other pathologies that involve endothelial dysfunction. In general, the interactions between endothelial cells, platelets, and other blood components are of great interest in a variety of diagnostics and therapeutics applications [23]. Our endothelial-cell-seeded model can be expanded to further explore such interactions and shed light on clotting mechanisms in the presence of various factors.

Here, we focused on recapitulating TTP, however, this platform can be expanded to recapitulate other pathologies that involve cell–platelet interactions. Additionally, this platform can be used to explore the effects of different drugs on clotting and offer new therapeutic prospects for various thrombolytic diseases.

Our microvascular network model was inspired by the cerebral microvasculature and represents a bifurcating microvascular network. Although the microvasculature is organ specific, and some organs’ microvasculature, such as the lungs, have a porous structure that differs from conventional branching [24], most microvascular networks are structured by bifurcating segments. Therefore, our cerebral-inspired network can be generalized to other networks with similar structures.

Due to the limitations of current 3D-printing technologies, our microvascular model is currently limited to a larger scale of 100–200 µm, which differs from human micro-capillaries’ dimensions. With advancements in 3D-printing technologies, the model can be further refined to match the micro-capillaries’ dimensions in the future.

An important drawback of our in vitro platform lies in the variability between patients. Since we used fresh human blood with different clotting patterns for each patient, as well as other differences in the blood composition between patients, the results varied significantly between donors. Additionally, it is possible that, in spite of our pre-query, patients were taking various undeclared drugs that affected the results. A standardization method should be developed to filter the donated blood prior to the experiment, and obtain a better understanding of phenomena, regardless of the specific donor conditions.

Nevertheless, our findings showed a clear relation between geometry, WSR, and clot formation, implying bifurcations as zones of facilitated thrombus formation. Additionally, this platform opens up avenues for numerous diagnostic and therapeutic applications, and may allow for tailoring specific treatments.

## Data Availability

The original contributions presented in the study are included in the article/Supplementary Materials, further inquiries can be directed to the corresponding author/s.

## Supplementary Materials

The following supporting information can be downloaded at: https://www.mdpi.com/article/10.3390/mi15080988/s1, Figure S1: Fluorescence intensity at the final clotting time point (t = 11 min) in collagen coated vascular injury models—straight vs. the Murray model microfluidic device. The bars present the average of the final fluorescence signal in 5 different patients. Significance was determined by unpaired Student’s *t*-test, * *p* < 0.05.
