# Supplementary material for: Clot Accumulation in 3D Microfluidic Bifurcating Microvasculature Network
Source: Micromachines (Basel). 2024 Jul 31;15(8):988. doi: 10.3390/mi15080988 (PMC11356079; doi:10.3390/mi15080988)
Supplement: Supplementary file 1 [file micromachines-15-00988-s001.zip › micromachines-3119551-supplementary.pdf]

Supplementary Information

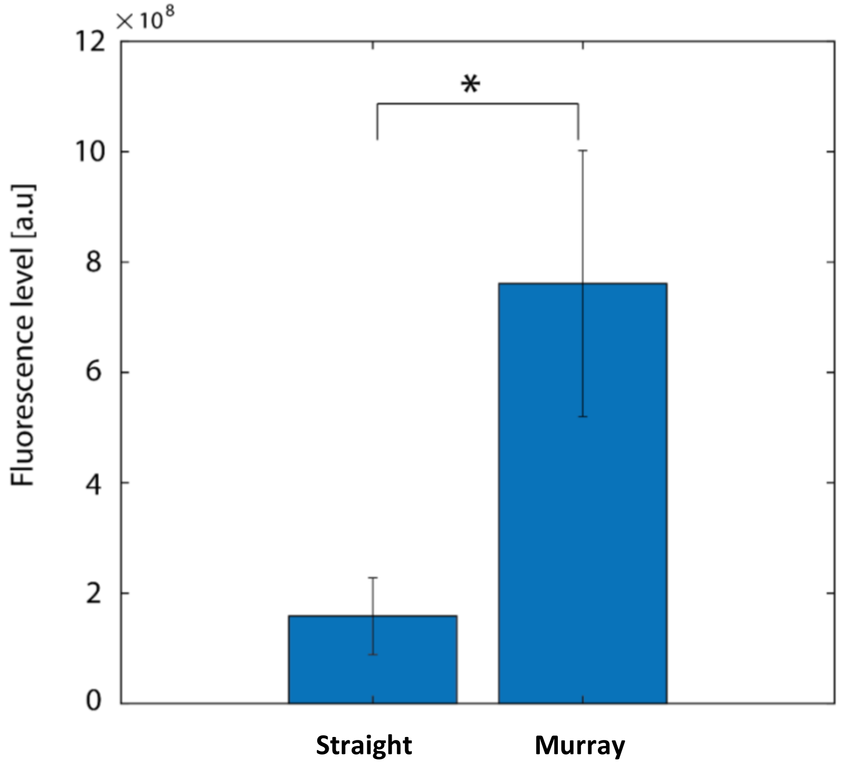

Figure S1. Fluorescence intensity at the final clotting time point (t= 11 min) in collagen coated vascular injury models – straight vs. the Murray model microfluidic device. The bars present the average of the final fluorescence signal in 5 different patients. Significance was determined by unpaired Student's t-test, * $p < 0.05$.
